# Analysis of Fatigue Property of the Aviation Gear Steel 15Cr14Co12Mo5Ni2 During High-Temperature Carburizing and Quenching

**DOI:** 10.3390/ma19102151

**Published:** 2026-05-20

**Authors:** Wei Feng, Yifan Zhou, Yuhao Zhang, Ruikun Wang, Xinhao Zhao

**Affiliations:** 1 School of Materials Science and Engineering, Wuhan University of Technology, Wuhan 430070, China; 13797323452@163.com (Y.Z.); 13966831013@163.com (Y.Z.); 18339541041@163.com (R.W.); 2 State Key Laboratory of Light Superalloys, Wuhan University of Technology, Wuhan 430070, China; 3 AECC Zhongchuan Transmission Machinery Co., Ltd., Changsha 410000, China

**Keywords:** 15Cr14Co12Mo5Ni2 aviation gear steel, austenitization temperature, axial tesion–compressive fatigue, fatigue life

## Abstract

15Cr14Co12Mo5Ni2, as a new type of low-carbon high-alloy aviation gear steel, has shown significant application potential in the transmission systems of aero engines due to its excellent high-temperature performance. In this paper, the aviation gear steel 15Cr14Co12Mo5Ni2 was treated by a carburizing and quenching process. The microstructure distributions of the carburized and quenched aviation gear steel at different austenitization temperatures (1020 °C, 1050 °C and 1080 °C) were analyzed by OM, SEM and EBSD. Subsequently, the axial tension–compressive fatigue tests (stress ratio R = −1) were carried out using a high-frequency fatigue testing machine after heat treatment at different austenitization temperatures, and the stress–number of cycles (S-N) curves were obtained by fitting the number of fatigue fracture cycles. The fracture morphologies were observed by SEM and the fracture mechanisms were analyzed. The research results show that the distribution of the microstructure and carbides exhibits gradient characteristics, and the carbide content decreases and the effective carburized layer depth decreases from 0.65 mm to 0.45 mm with increasing austenitization temperature, and the main carbide types are M_23_C_6_ and M_7_C_3_. The fatigue life of 15Cr14Co12Mo5Ni2 gear steel decreases as the austenitization temperature increases. Within the selected temperature range of 1020 °C, 1050 °C, and 1080 °C in this study, the fitted fatigue strengths at a given fatigue life of 10^6^ cycles are 192 MPa, 183 MPa, and 158 MPa, respectively. No obvious crack initiation site can be directly observed from the fracture morphologies of all specimens. Based on the characteristics of crack propagation, it is inferred that the crack source is located in the core or near-core region, and the cracks propagate outward from the core and the propagation rate accelerates with the increasing austenitization temperature, eventually fracturing in the carburized layer. The fracture mechanism of 15Cr14Co12Mo5Ni2 gear steel at the austenitization temperatures of 1020 °C was a mixed mode of intergranular and cleavage brittle fracture, while at 1050 °C and 1080 °C, it was mainly brittle fracture accompanied by local ductile fracture.

## 1. Introduction

Aviation gears typically operate in extreme service environments such as high speeds above 20,000 r/min, elevated temperatures above 200 °C, and alternating heavy loads above 3 GPa, and are required to possess high load-bearing capacity and fatigue resistance to ensure the normal operation of aero engines and the safe flight of airplanes [1,2]. In most cases, fatigue failure is the main failure mode of aviation gears. Fatigue failure occurs when the stress level is far lower than the static tensile strength of the material and suddenly leads to fracture after a sufficient number of cycles such as the high cycle fatigue regime of about 10^5^–10^7^ cycles and the very high cycle fatigue above 10^7^, which is characterized by its concealment and often results in catastrophic consequences [3,4]. Therefore, fatigue failure has become a persistent core challenge due to its inherent complexity and unpredictable features in engineering design and materials science, especially for aviation gear components and materials serving in extreme environments [5].

Fatigue performance is not an intrinsic material property, but rather a characteristic that exhibits high sensitivity to microstructure variations such as grain type, size, distribution, and so on [6,7,8]. This characteristic provides a basis for actively regulating the microstructure to improve the fatigue strength, hence enhancing the service life of components. Among available methods for controlling microstructure, heat treatment plays a crucial role in shaping the final performance of materials. Studies have shown that rational design of heat treatment parameters can effectively enhance the fatigue resistance of materials [9,10,11,12]. Surface hardening treatments techniques, such as carburizing, nitriding and induction hardening, are widely used on low carbon alloy steels by changing the surface structure and stress state to improve their fatigue resistance [13,14,15,16,17]. Quenching is a fundamental heat treatment process that significantly enhances the mechanical properties of materials by generating metastable, non-equilibrium microstructures. During the quenching process, the austenitization temperature before quenching acts as the governing parameter, functioning as a precise initiator for microstructural evolution, phase transformations, dissolution and precipitation of alloying elements and residual stress and deformation, which regulates the material’s hardness, strength and resultant toughness. However, the relationship between strength and fatigue performance is not an oversimplified positive correlation. An increase in austenitization temperature before quenching can enhance the hardness and strength of materials, but its fatigue strength will decrease [18,19].

In the heat treatment process of alloy steel, the austenitization temperature will significantly affect the grain size of austenite, the solubility of carbon and alloy elements, and their distribution in the matrix, thus influencing the mechanical properties. During heating 52100 bearing steel, Li et al. [20] found that increasing the austenitization temperature will reduce the volume fraction of undissolved carbides and increase the carbon content, thus leading to the conflict between hardness and toughness. It was found that the grain size of austenite increases from 25.4 μm to 52.8 μm when the temperature increases from 1150 °C to 1250 °C during the austenitizing treatment of 700 MPa grade high-strength steel [21]. Increasing the austenitizing temperature above 1050 °C promotes the dissolution of coarse M23C6-type carbides during heat treatment of 10Cr12Ni3Mo2VN steel, resulting in improved low-temperature toughness [22]. When the austenitizing temperature is too low, the austenitization is incomplete, and the carbonides are not fully dissolved, resulting in the formation of the undissolved hard phase, which becomes a crack source for the early initiation of fatigue cracks. When the austenitizing temperature is too high, the austenite grains will undergo rapid coarsening, thus reducing the material’s strength.

By choosing an appropriate austenitization temperature before quenching for metallic materials, a finer ferrite structure or martensite-bainite multiphase structure can be obtained, and dislocation slip can also be delayed, thereby enhancing the fatigue performance of the material [18,23]. This is because bainite can modulate the strength and toughness balance of the material, and higher toughness is conducive to hindering the propagation of microcracks, thereby potentially improving fatigue performance [24]. Additionally, some research indicates that austenitizing temperature will affect the content and stability of residual austenite for the quenched steel sample. As we all know, the retained austenite was metastable and the stability of its mechanical properties will change with temperature variations, and it will partially or completely transform into a martensitic structure under strain-induced action [25,26]. Appropriate and uniformly distributed residual austenite is conducive to the good fatigue performance of the material due to the transformation of RA will be beneficial to reduce the rate of crack propagation [27]. For low-carbon steel that has undergone carburizing and quenching treatment, the transformation of retained austenite into martensite will lead to instability and anisotropy of residual stress during cyclic loading, thereby affecting the fatigue performance of the material [28]. Excessive or unstable residual austenite may prematurely transform under cyclic stress, instead introducing microcracks or causing dimensional instability. Meanwhile, the austenitization temperature before quenching directly determines the residual stress state within the material after quenching and cooling. After quenching and tempering treatment of high-carbon 52100 steel, the higher initial amounts of retained austenite will improve fatigue crack growth resistance and inhibit brittle intergranular fracture and promote transgranular fracture. This might be due to the transformation of RA near crack propagation which will intensify the compressive stress at the crack tip and form microcracks at the untempered martensite interface [29]. The surface residual compressive stress is recognized as a favorable factor that can effectively close fatigue cracks and enhance fatigue strength (especially high-cycle fatigue) [30,31,32].

15Cr14Co12Mo5Ni2 is a new generation of low-carbon and high-alloy aviation gear steel. By determining the appropriate carburizing and quenching heat treatment process, the alloy can obtain high surface hardness and better core fracture toughness and impact resistance, which can meet the high temperature, high load and impact working conditions of aero engines [33]. At present, several scholars have studied its high-temperature fatigue strength after carburizing and quenching treatment [34], as well as the evolution mechanism of microstructure and mechanical properties after cold forging forming [35]. However, no studies have reported the influence of the austenitization temperature before quenching on the fatigue behavior of this alloy yet.

In the present work, the vacuum carburizing and high-temperature quenching heat treatment process were carried out on 15Cr14Co12Mo5Ni2 aviation gear steel. To understand and evaluate the effect of quenching temperature on fatigue resistance, the axial tension–compressive fatigue tests were performed on a high-frequency fatigue testing machine at different austenitization temperatures (1020 °C, 1050 °C and 1080 °C). The stress–number of cycles (S-N) curves were obtained by fitting the fatigue test data. The fracture morphologies were observed by SEM and the fracture mechanism was analyzed. This research can provide a theoretical basis and experimental support for the process parameter optimization of 15Cr14Co12Mo5Ni2 steel in the manufacturing of high-performance and long-life aviation gears.

## 2. Materials and Methods

### 2.1. Material Preparation and Heat Treatment Process

The aviation gear steel 15Cr14Co12Mo5Ni2 ingot used in this work was smelted by the vacuum induction melting + vacuum arc remelting (VIM + VAR) method and forged into round bars, and its main chemical composition is shown in Table 1.

**Figure 1:**
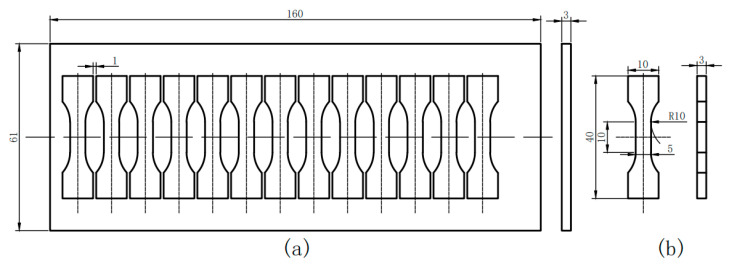
Sample diagram. (**a**) heat-treated sample; (**b**) fatigue test specimens (unit: mm).

**Figure 2:**
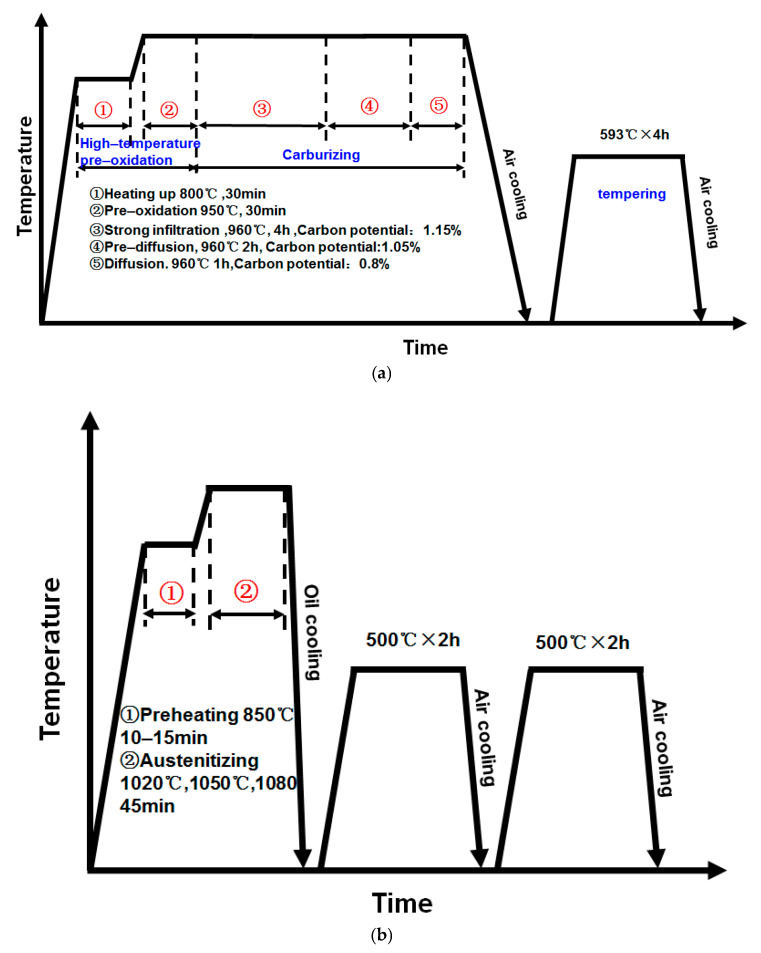
Schematic diagram of heat treatment process: (**a**) carburizing and high-temperature tempering stage; (**b**) quenching and tempering stage.

Three aviation gear steel plate samples with a length of 160 mm, a width of 60 mm and a thickness of 3 mm were cut from a round bar annealed along a direction parallel to the rolling direction for heat treatment experiments, as shown in Figure 1a. Then the plate samples underwent heat treatment according to the process as shown in Figure 2 [35]. The heat treatment process can be divided into two parts. The first part is the carburizing and high-temperature tempering stage: The vacuum carburizing furnace was first heated to 800 °C, with a holding time of 30 min. Then, the temperature rises to 950 °C holding for 20 min for high-temperature pre-oxidation. Subsequently, carburizing was carried out at 960 °C under different carbon potentials (1.15%, 1.05% and 0.8%, respectively) for 7 h, followed by air cooling, and then, high-temperature tempering was carried out at 593 °C for 240 min and air cooling, as illustrated in Figure 2a. After carburizing in a vacuum furnace, the quenching and tempering stage was performed: three plate samples were heated at 1020 °C, 1050 °C, and 1080 °C respectively for 45 min and then quenched by oil, followed by tempering at 500 °C for 2 h, air cooling, finally by supplementary tempering at 500 °C for 2 h, air cooling, as shown in Figure 2b.

### 2.2. Fatigue Experiments

**Figure 3:**
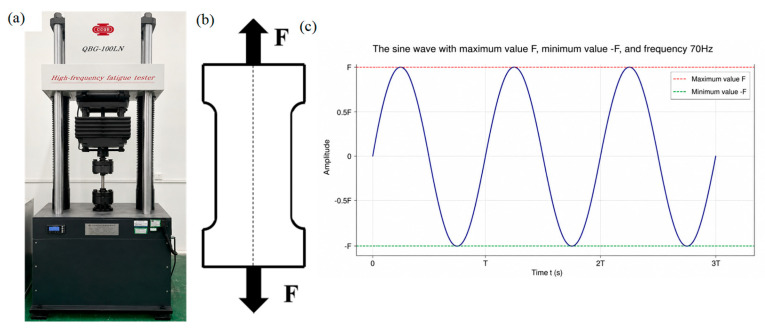
High-frequency fatigue testing machine. (**a**) The physical specimen of the testing machine; (**b**) load model; (**c**) load condition.

Twelve fatigue samples were taken from each steel plate treated at different austenitizing temperatures at intervals of 1 mm, as shown in Figure 1a. The specimen shape and specific dimensions with a gauge length of 10 mm are illustrated in Figure 1b. The samples used for fatigue test were slightly ground in sequence by 800#, 1000# and 2000# SiC sandpapers, particularly attention paid to the gauge section during grounding. The gauge section should be kept flat, smooth and dimensionally uniform, and defects produced by wire cutting such as burrs, sharp edges, and surface scratches should be removed to ensure the specimen geometry, dimensional accuracy and surface quality to meet the requirements for axial tension–compression fatigue testing. The tension–compression fatigue test was conducted on the QBG-100LN high-frequency fatigue testing machine, as shown in Figure 3a. The cyclic load F was applied along the centerline of the sample to simulate the stress state under working conditions, as depicted in Figure 3b. A constant stress ratio of R =-1 was used, and the loading amplitude was controlled by applying a sine waveform with a frequency of 70 Hz. To obtain the stress versus cycles to failure (S-N) curve, cyclic load F can be calculated by adjusting the stress amplitude according to Equation (1):

(1) F=σ×B×H

where σ represents the stress amplitude. B is the sample width, which is 10 mm, and H represents the sample thickness, which is 3 mm.

In this experiment, the stress level is set to be related to the yield strength σ_0_ of the aviation gear steel, they are 80%, 60%, 40% and 20% of the yield strength σ_0_ respectively. In the previous tests, the yield strength σ_0_ was measured to be 1016 MPa [36]. Therefore, during fatigue tests, the stress amplitudes are set to 812 MPa, 609 MPa, 406 MPa and 203 MPa, respectively, and the samples are subjected to the cycle loading as shown in Figure 3c under different stress amplitudes. During fatigue tests, the run-out criterion is set to 10^6^ cycles, which means the test will stop if the sample does not fail before the number of cycles exceeds 10^6^ under the given stress. In order to ensure the accuracy of the experiment results, three repeated fatigue tests were performed at every stress level under different austenitizing temperatures. The total number of specimens prepared is 36, and 36 fatigue tests were carried out.

### 2.3. Microstructure Characterization

After the fatigue experiments, the fatigue fracture surfaces and the microstructures of the specimens after heat treatment at different austenitizing temperatures were observed and analyzed using an optical microscope (OM, IE500M, SUNNY GROUP, Ningbo, China), scanning electron microscope (SEM, JSM-IT800, JEOL, Tokyo, Japan) operated at 15 kV, energy dispersive spectrometer (EDS, attached to the JSM-IT800, JEOL, Tokyo, Japan) and electron back scattered diffraction (EBSD). EBSD analysis was performed using a Zeiss Gemini 300 (Carl Zeiss AG, Oberkochen, Germany) field-emission scanning electron microscope equipped with an Oxford Canon EBSD system at an accelerating voltage of 20 kV, with a scanning area of 32 × 24 μm^2^ and a step size of 0.05 μm. Prior to OM and SEM observations, the specimens were ground in sequence by 80#, 800#, 1000# and 2000# SiC sandpapers, followed by polishing with a 2.5 μm diamond suspension. Subsequently, the polished specimens were ultrasonically cleaned and then etched for approximately 2 min in a solution consisting of HCl, HNO_3_, CuCl_2_, FeCl_3_, H_2_O and anhydrous ethanol. Before EBSD examination, the specimens were further prepared using an IM4000plus argon ion polishing instrument (Hitachi High-Tech Corporation, Tokyo, Japan) at a voltage of 4 kV, the incident angle of 3° for 2 h in the first stage, followed by 2.5 kV, 1.5° and 1.5 h in the second stage.

### 2.4. Hardness Tests

The Vickers hardness of the heat-treated samples was measured by using the Huayin Micro Vickers hardness tester (HV-1000A, Laihua Testing Instrument Co., Ltd., Hangzhou, China). During the testing process, the starting position for hardness measurement was set at a distance of 0.05 mm from the cross-section of the sample. The hardness test was conducted from the surface inward at intervals of 0.1 mm along the rolled direction with a load of 200 g for 10 s. Three different positions were selected for hardness testing, and the average value of each measurement was taken to ensure the accuracy of the Vickers hardness measurement.

## 3. Experimental Results and Discussion

### 3.1. Microstructure Characteristics

**Figure 4:**
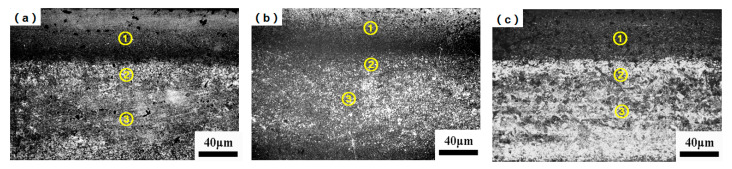
OM diagrams after heat treatment at different austenitization temperatures: (**a**) 1020 °C; (**b**) 1050°C; (**c**) 1080°C. ①, ② and ③ indicate the carburized zone, the transition zone and core zone, respectively.

The optical micrographs of the aviation gear steel 15Cr14Co12Mo5Ni2 from the surface to the core at different austenitization temperatures after carburizing and quenching treatment are illustrated in Figure 4. It can be observed that all the samples at the three temperatures present three typical regions: the outer high-carbon carburized zone ①, the middle low-carbon transition zone ②, and core zone ③ without carbon enrichment in the center.

**Figure 5:**
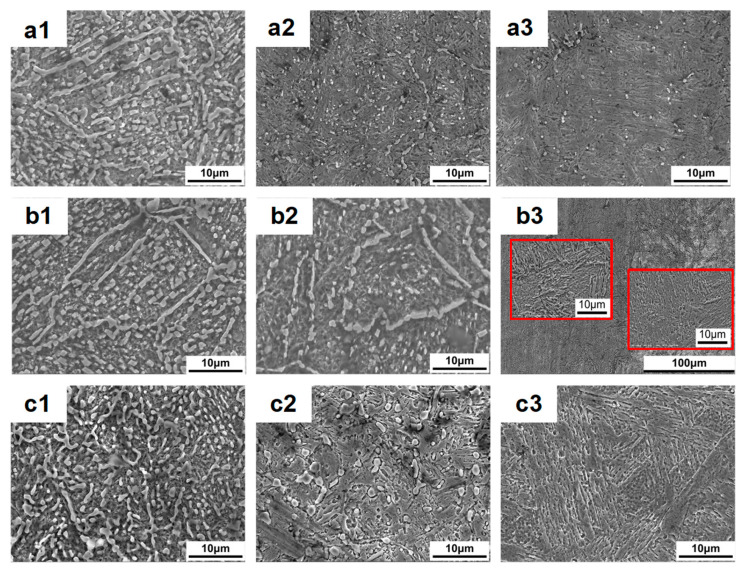
SEM images of the microstructure distribution in the carburized zone (**a1**–**c1**), transition zone (**a2**–**c2**) and core zone (**a3**–**c3**) of the sample at different austenitization temperatures: (**a1**–**a3**) 1020 °C; (**b1**–**b3**) 1050 °C; (**c1**–**c3**) 1080 °C. Red squares in the figure b3 are the magnification graph at this position.

Figure 5 shows the scanning electron microscope (SEM) morphologies of the carburized zone, transition zone and core zone of the sample at different austenitization temperatures. From Figure 5(a1,b1,c1), it can be found that a large amount of carbides are observed in the carbide layer and are mainly distributed in a continuous network and chain pattern along the grain boundaries as well as in granular form within the grains at all austenitization temperatures. In the transition zone, the carbides are fine in size and dispersed in the matrix at the austenitization temperature of 1020 °C. The amount of carbides decreases, but their distribution pattern is similar to that of the carburized layer at the austenitization temperature of 1050 °C. When heated to the austenitization temperature of 1080 °C before quenching, the carbides tend to coarsen and have irregular contours, as shown in Figure 5(a2,b2,c2). As seen from Figure 5(a3,b3,c3), the austenitization temperature before the quenching has a significant influence on the microstructure of core zone: at 1020 °C, a small amount of fine-sized carbide particles are dispersed in the matrix; at the austenitization temperature of 1050 °C, two distinct microstructure regions are observed and lath martensites of different size and spacings are observed by high-magnification scanning electron microscope; at the austenitization temperature of 1080 °C, only a small number of fine carbides remain, and the uniformity of the microstructure has been significantly improved.

In summary, as the austenitization temperature before quenching increases, the solubility of carbides in austenite continuously increases, resulting in a gradient evolution of the carbon content, morphology, size, and spatial distribution of carbides from the surface to the core region.

**Figure 6:**
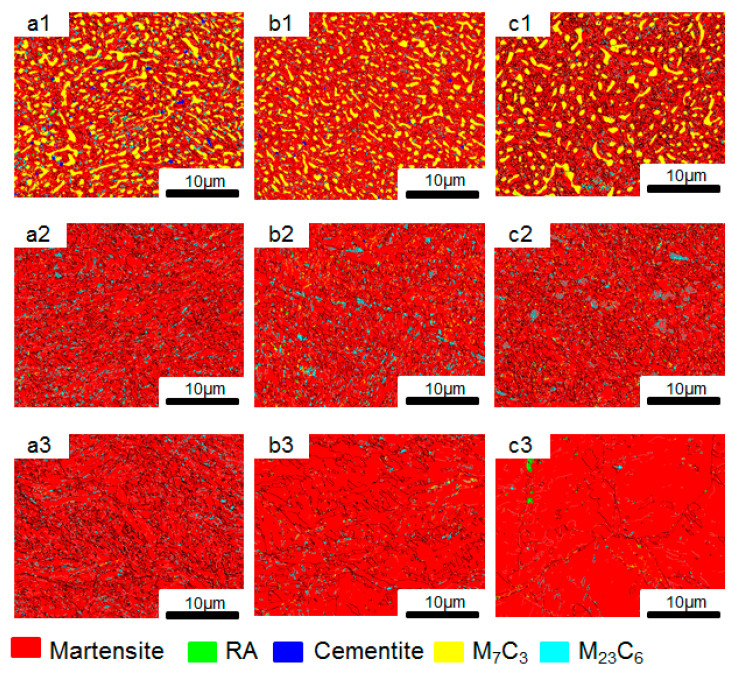
EBSD images of phase distribution and grain boundary superposition in the carburized zone (**a1**–**c1**), transition zone (**a2**–**c2**) and core zone (**a3**–**c3**) of the sample at different austenitization temperatures: (**a1**–**a3**) 1020 °C; (**b1**–**b3**) 1050 °C; (**c1**–**c3**) 1080 °C.

Figure 6 shows the EBSD phase distribution and grain boundary superposition diagrams of samples at different temperatures. Through the phase diagrams, the distribution and content of each phase in different regions at different temperatures can be determined.

It can be seen from Figure 6 that the matrix structure of the sample mainly transforms into a martensitic phase with a BCC structure after heat treatment. In the carburized zone, the main carbide types are M_23_C_6_ and M_7_C_3_, among which the content of M_7_C_3_-type carbides is relatively high and mainly distributed along the grain boundaries. The M_23_C_6_-type carbides have a relatively low content and are mainly distributed within the grain boundaries. In the transition zone, the carbon content decreases and the content of M_23_C_6_-type carbides increases, while the content of M_7_C_3_ decreases significantly. As the carbon content in the core zone further decreases, the matrix contains a small amount of M_23_C_6_-type carbides at temperatures of 1020 °C and 1050 °C.

Additionally, it can also be observed from Figure 6 that the grain boundary distribution was relatively dense for the specimen austenitized at 1020 °C, whereas it became relatively sparse for the specimen austenitized at 1080 °C, which suggests martensitic lath structure tends to gradually coarsen with increasing austenitization temperature.

Table 2 shows the content of each phase in different regions at different austenitization temperatures. As can be seen in Table 2, the total content of carbides in the carburized layer gradually decreases with the increase in austenitization temperature, among which M_7_C_3_ gradually decreases and M_23_C_6_ gradually increases. The content of martensite increases with the rise in temperature. It can also be known from Table 2 that there is a very small amount of residual austenite (RA) in the matrix after heat treatment.

### 3.2. Hardness Distribution

**Figure 7:**
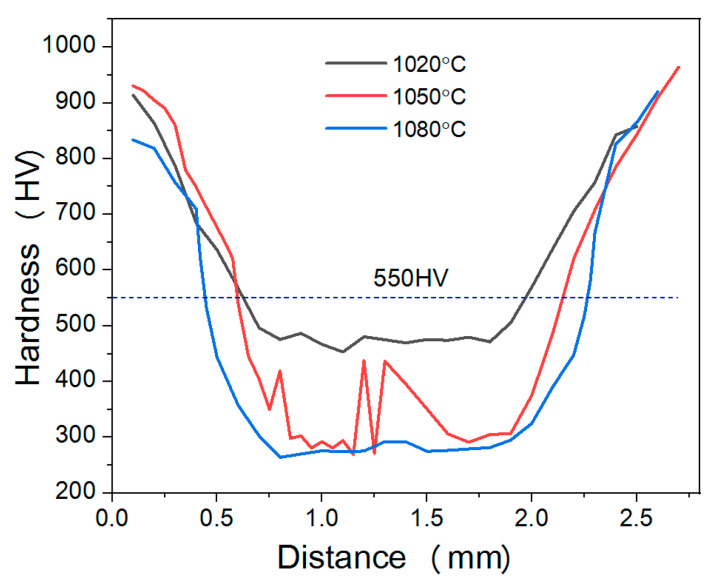
Hardness variation curves from the carburized layer to the core at different austenitization temperatures.

Figure 7 shows the hardness variation curves from the carburized layer to the core at different austenitization temperatures. It can be observed from Figure 7 that the hardness of the samples at the three different temperatures all exhibits a gradient distribution characteristic, gradually decreasing from the surface to the core. The surface hardness of the sample is the highest, and it progressively decreases as the distance towards the core increases, eventually reaching a relatively low and stable level in the core region.

After austenitizing at 1020 °C and 1050 °C, the sample surfaces have high hardness levels, with peak hardness values approximately ranging from 900 to 950 HV. However, for the sample austenitized at 1080 °C, the surface hardness slightly decreased, approximately 830–850 HV. In the transition zone from the surface to the core, the hardness also varied with the austenitization temperature. The hardness of the specimens austenitized at 1020 °C decreases relatively gently, with a wider transition zone. However, the hardness of the specimens austenitized at 1080 °C decreases more steeply, and the transition zone is relatively narrow. The hardness of the sample austenitized at 1050 °C showed intermediate behavior between the two temperatures.

The effect of different austenitization temperatures on core hardness was significant. The core hardness of the samples austenitized at 1020 °C is the highest and changes little, with an average value of approximately 470–490 HV, indicating that the core microstructure is relatively uniform. The sample austenitized at 1080 °C exhibited a generally lower core hardness, averaging around 270–290 HV, suggesting that although high-temperature austenitization made the core microstructure more continuous, the coarsening of the phase structure reduced hardness. In contrast, the core hardness distribution of the samples austenitized at 1050 °C is uneven, with an average hardness value of approximately 300–330 HV, reflecting poor uniformity in the core microstructure.

According to ISO 2639 [37], the case-hardened depth of the sample after carburizing and quenching is generally defined as the perpendicular distance from the surface to the layer with a hardness of 550 HV. Therefore, 550 HV was adopted to determine the effective carburized layer depth in the present study. Based on the average hardness distribution curve in Figure 7, defining a hardness of 550 HV as the effective carburized layer depth, it can be determined that the effective carburized layer depths of the samples austenitized at 1020 °C, 1050 °C and 1080 °C before quenching are approximately 0.65 mm, 0.60 mm and 0.50 mm, respectively, which indicates that the effective carburized layer depth gradually decreases with the austenitization temperature increasing.

### 3.3. Fatigue Performance Test Results

Table 3 presents the stress–number of cycles (S–N) fatigue test results for untreated initial specimens and those treated at different austenitization temperatures, where run-out indicates that the specimen does not fail after exceeding 10^6^ cycles. Since the fatigue test data are usually discrete, statistical methods are generally required to fit these experimental results to obtain the S–N curve. Typically, the power function shown in Equation (2) is adopted for fitting the fatigue test results [38,39].

S^m^N = C (2)

where S represents stress level, N represents the number of cycles under the current stress level, and m and C represent material characteristic parameters.

During the S–N curve fitting, the data that showed run out should be treated as right-censored observations rather than failure data according to the ISO standard [40]. Therefore, they were not assigned an artificial failure life of 10^6^ cycles and were not included in the regression fitting of Equation (2). Only the specimens with definite fatigue fracture cycles were used for fitting the S–N curves. Specifically, for the specimen austenitized at 1020 °C before quenching under 203 MPa, only the failed specimen with N_f_ = 5.537 × 10^5^ cycles was included in the fitting, while data from the other two run-out specimens were plotted only as censored data. For the specimens austenitized at 1050 °C and 1080 °C before quenching under 203 MPa, all three specimens were run outs; therefore, the S–N data at the stress level were only marked as run-out data in the S–N plots. Considering that only three specimens were tested at each stress amplitude, a statistical scatter analysis was performed based on the existing fatigue life data. Since fatigue life usually exhibits logarithmic discrete characteristics, the fatigue life of failed specimens was first converted into logarithmic form according to Equation (3) [40,41], and its standard deviation (SD) was calculated to analyze the statistical scatter.

x_i_ = lg(Nf*_i_*) (3)

where Nf*_i_* is the number of cycles to failure of the *i*-th specimen.

**Figure 8:**
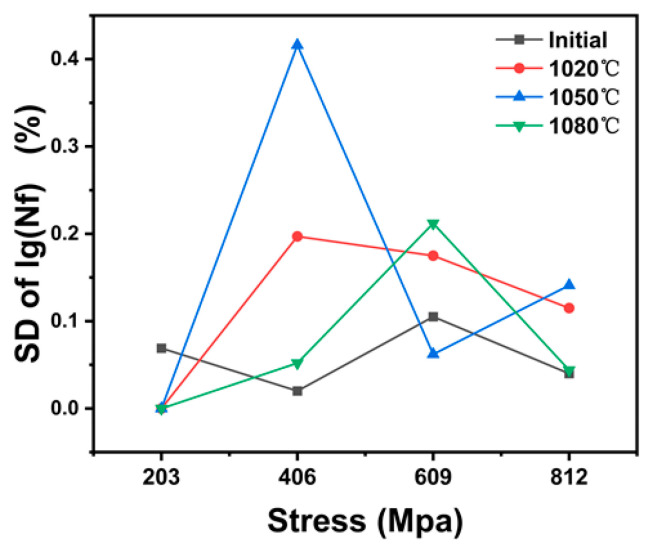
Variation curve for SD of lgNf under different stress amplitudes.

Run-out data at 10^6^ cycles were treated as right-censored data and were not included in the statistical calculation. The calculated standard deviation (SD) of the logarithmic form of the fatigue life of failed specimens under different temperature and different stress amplitudes are shown in Figure 8. The results show that the SD, the scatter degree of fatigue life, varies with stress amplitude and heat-treatment condition, and their values are smaller, which means the dispersion degree of fatigue life data is relatively small.

**Figure 9:**
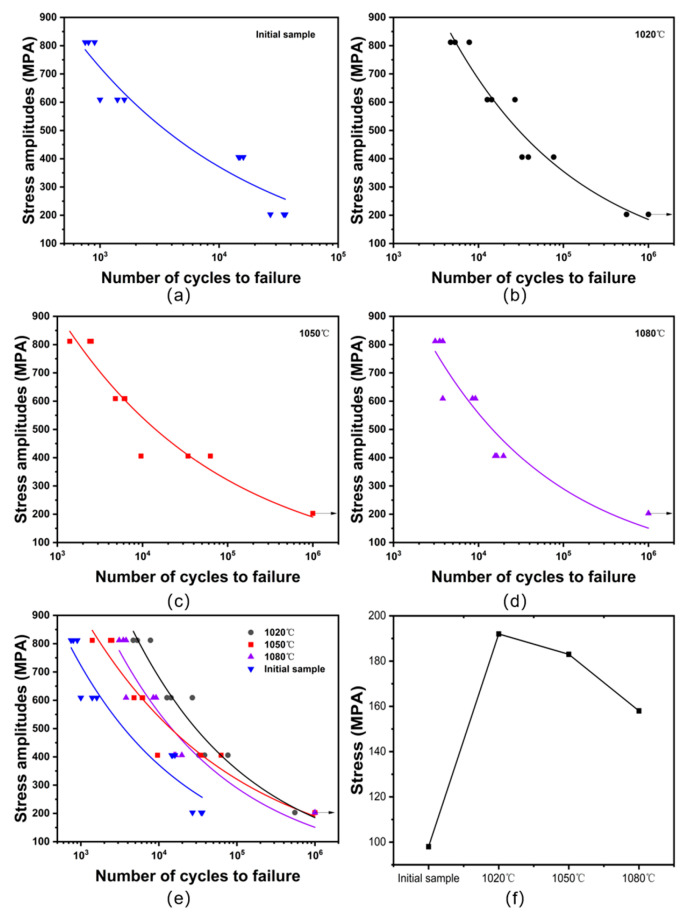
S–N curves at different conditions: (**a**) the initial sample; (**b**) at the austenitization temperature of 1020 °C; (**c**) at the austenitization temperature of 1050 °C; (**d**) at the austenitization temperature of 1080 °C; (**e**) S–N curves at different conditions; (**f**) the fatigue strength at different conditions at 10^6^ cycles.

Substituting the fracture failure data in Table 3, except for the right-censored run-out observations, into Equation (2), the fitting S–N curves are obtained as shown in Figure 9. The adjusted coefficients R^2^ values of the fitted curves for the fatigue data of each specimen are relatively high, which are 0.91, 0.95, 0.94, and 0.91, respectively. This indicates that the S–N curve fitting accuracy for each group of specimens is high, and all curves can effectively characterize the experimental data.

By analyzing the life data at each stress level in Table 3 and Figure 9, it can be found that the life of the sample has significantly increased after heat treatment. The fatigue life of the specimens after heat treatment decreased significantly with the increase in stress level. The fatigue life of the original specimens ranged from 2.7 × 10^4^ to 3.6 × 10^4^ cycles at a low stress level of 203 MPa and further decreased to a range of 7.5 × 10^2^ to 9.0 × 10^2^ cycles at a high stress level of 812 MPa, exhibiting the poorest fatigue resistance, as shown in Figure 9a.

After the specimens were austenitized at 1020 °C before quenching, at a low stress level of 203 MPa, two specimens did not fracture at 10^6^ cycles, and the fatigue life of the other specimen reached 5.537 × 10^5^ cycles. At a moderate stress level of 406 MPa, the fatigue life range is 3.26 × 10^4^ to 7.68 × 10^4^ cycles. At a relatively high stress level of 609 MPa, the fatigue life remains between 1.27 × 10^4^ and 2.69 × 10^4^ cycles, while at a high stress level of 812 MPa, the fatigue life decreases to 4.70 × 10^3^ to 7.80 × 10^3^ cycles, as shown in Figure 9b.

After austenitizing at 1050 °C, all three specimens exhibited run out at a low stress level of 203 MPa. When the stress level increased to 406 MPa, the fatigue life decreased to the range of 9.60 × 10^3^ to 6.27 × 10^4^ cycles, showing considerable scatter. At a relatively high stress level of 609 MPa, the specimen life ranged from 4.80 × 10^3^ to 6.20 × 10^3^ cycles. However, when the stress increased to a high level of 812 MPa, the fatigue life decreased to a range of 1.40 × 10^3^ to 2.50 × 10^3^ cycles, as shown in Figure 9c.

Figure 9d shows the S–N diagram of the austenitization temperature of 1080 °C. Similar to specimens quenched at 1050 °C, run out occurred in all three specimens at a low stress level of 203 MPa. At a medium stress level of 406 MPa, the life ranged from 1.57 × 10^4^ to 1.97 × 10^4^ cycles. At a relatively high stress level of 609 MPa, the fatigue life is between 3.80 × 10^3^ and 9.20 × 10^3^ cycles. When the stress increased to a high level of 812 MPa, the life was reduced to a range of 3.10 × 10^3^ to 3.80 × 10^3^ cycles.

From Figure 9e, it can be known that at a low stress level of 203 MPa, increasing the austenitization temperature is beneficial for prolonging the fatigue life of the sample. However, at relatively high stress levels, raising the austenitization temperature will instead lead to a reduction in fatigue. Under the condition of a constant austenitization temperature, a slight increase in stress will decrease the fatigue life of the sample, indicating that the sample is sensitive to stress changes.

Figure 9f illustrates the fatigue strength of the original specimens and the specimens treated at different austenitization temperatures at a given fatigue life of 10^6^ cycles according to the S–N curve obtained by fitting the fatigue test data. It can be obtained that the predicted fatigue strengths are 98 MPa, 192 MPa, 183 MPa and 158 MPa, respectively. The fatigue strengths at 10^6^ cycles obtained from the fitted S–N curves should be regarded as fitted estimates rather than statistically established fatigue limits. Consequently, the high-cycle fatigue performance of the specimens after heat treatment was significantly improved compared with the original specimens, and it decreased with the increase in austenitization temperature according to the fitting result. The fitted fatigue strength of the specimens is relatively higher at the austenitization temperature of 1020 °C before quenching.

### 3.4. The Effect of Austenitization Temperature on Fatigue Life

Based on the above analysis, it is evident that the fatigue performance of the heat-treated specimens is significantly superior to that of the original specimens. The main reason is that after carburizing and quenching treatment, the aviation gear steel forms a gradient microstructure from the surface to the core. The high-hardness carburized layer on the surface enhances the resistance to crack initiation, and the transition zone mitigates the property mismatch between the surface and the core, while the core provides good toughness.

The high-cycle fatigue performance of the aviation gear steel decreases with increasing austenitization temperature, and the fatigue strength of the specimens is relatively higher at the austenitization temperature of 1020 °C within the selected three temperature range in this work. This is mainly because the increase in austenitization temperature will accelerate the coarsening and agglomeration of carbides in the carburized layer, promoting the refinement and dissolution of carbides in the core. At the austenitization temperature of 1020 °C, the carbides in the carburized layer are relatively abundant, fine, and uniformly distributed within the matrix, while the martensite in the core is finer and less abundant compared to the other two austenitization temperatures.

Furthermore, as the austenitization temperature increases, the average surface hardness first increases and then decreases, while the depth of the effective carburized layer gradually reduces. At 1020 °C, the specimen surface exhibits relatively high hardness and sufficient case depth, which can effectively raise the threshold for surface crack initiation. Additionally, its core hardness is the highest with minimal fluctuation, indicating that the core microstructure is more uniform and stable. Therefore, although the surface layer of the sample austenitized at 1020 °C does not have the highest hardness, the synergistic effect of surface crack resistance and core load-bearing capacity is the strongest, resulting in the highest high-cycle fatigue life.

Although the specimen austenitized at 1050 °C before quenching achieves the highest surface hardness, its overall fatigue performance remains inferior to that at 1020 °C, indicating that fatigue performance is not solely governed by surface hardness, but is also strongly affected by the matching relationship among the hardened carburized layer, transition zone and load-bearing core. Combining SEM, EBSD, and hardness results reveals that the specimen austenitized at 1050 °C has an uneven microstructure in the core zone and the hardness from the surface to the core fluctuates greatly. This means that under cyclic loading, localized deformation incompatibility and stress concentration are more likely to occur in the core and transition zone. Once cracks initiate in such localized weak regions, the advantage of high surface hardness is insufficient to translate into an overall life advantage.

For the specimen austenitized at 1080 °C before quenching, the core is more fully austenitized and has fewer residual carbides, but its surface hardness decreases, the depth of the effective hardened layer reduces, and the core hardness also declines. In other words, although high-temperature quenching improves the microstructure continuity in some areas, it weakens the dual effect of the gradient microstructure for surface strengthening and core toughness, ultimately resulting in lower fatigue strength and life compared to the specimens austenitized at 1020 °C and 1050 °C.

In summary, the fatigue performance of the heat-treated specimens is significantly superior to that of the original specimens. The effect of austenitization temperature before quenching on fatigue behavior should be understood as a coupled response of carbide morphology, effective case depth, hardness gradient, and microstructural matching between the carburized layer and the core. Within the studied temperature range in this paper, the specimen austenitized at 1020 °C before quenching exhibited a relatively favorable coordination in the effective case depth, relatively uniform core hardness, finer microstructural features and gradient structural matching. Therefore, the fitted S–N results suggest a comparatively higher fatigue strength at a given fatigue life of 10^6^ cycles. For the specimen austenitized at 1050 °C before quenching, although the surface hardness was relatively high, the heterogeneous core microstructure and hardness fluctuation may promote local deformation incompatibility under cyclic loading, which weakens the overall fatigue life and shows relatively scattered fatigue life. For the specimen austenitized at 1080 °C before quenching, the reduced effective case depth, lower surface hardness and coarser microstructural features may result in the lower fitted fatigue strength at a given fatigue life of 10^6^ cycles.

### 3.5. Fatigue Morphology and Fracture Mechanism Analysis

The fracture failure process of metallic materials mainly includes three stages: crack initiation, crack propagation and fracture failure. Under the action of cyclic loading, the damage caused by stress concentration on the surface and near the surface of the material will form crack sources, thereby leading to the initiation of fatigue cracks. By observing the macroscopic fracture morphology of the fractured specimen, the location of the fracture source and the fracture mode of the specimen can be determined. Then, the fracture mechanism of the sample was further analyzed by observing the typical fracture source locations through SEM tests.

**Figure 10:**
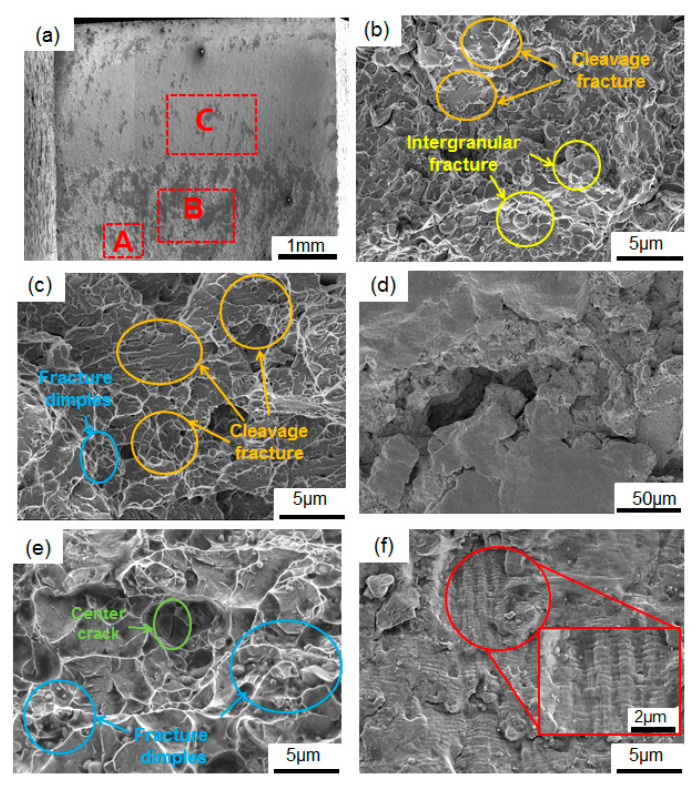
Fracture morphology of samples at the austenitization temperature of 1020 °C with 406 MPa (N_f_ = 3.86 × 10^4^): (**a**) macroscopic morphology; (**b**) the carburized layer; (**c**,**d**) the transition layer; (**e**) the core; (**f**) fatigue striations. A B and C indicate the SEM observation site of the carburized layer, the transition layer and core zone, respectively.

**Figure 11:**
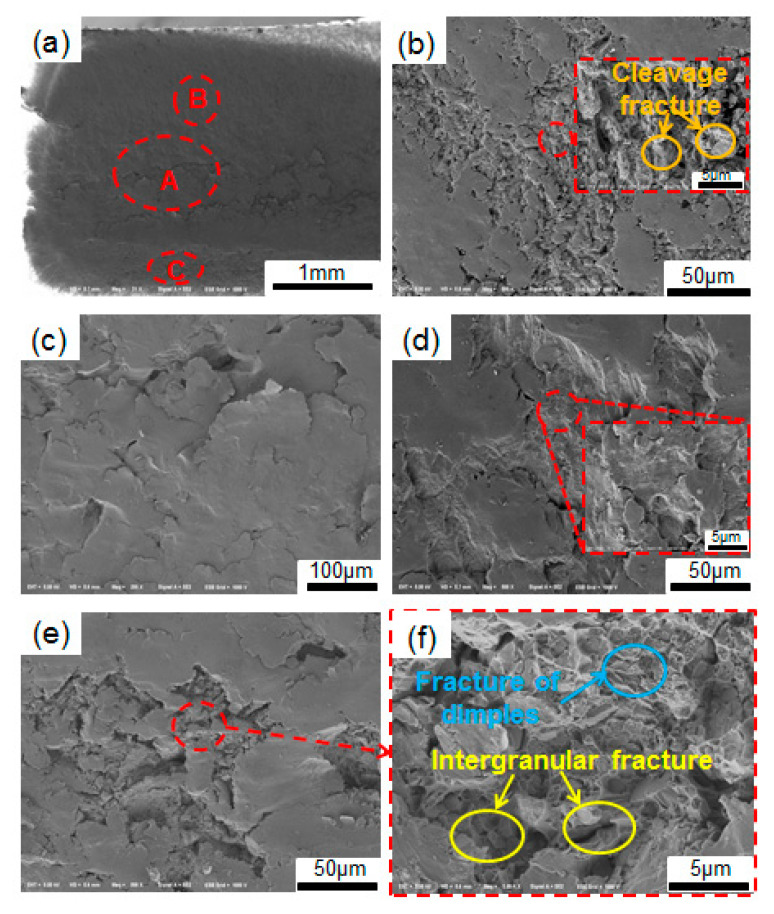
Fracture morphology of samples at the austenitization temperature of 1050 °C with 406 MPa (Nf = 3.43 × 10^4^): (**a**) macroscopic morphology; (**b**,**c**) the core; (**d**) the transition layer; (**e**) the carburized layer; (**f**) high-magnification map of (**e**). A B and C indicate the SEM observation site of the core, the transition layer and carburized layer, respectively.

**Figure 12:**
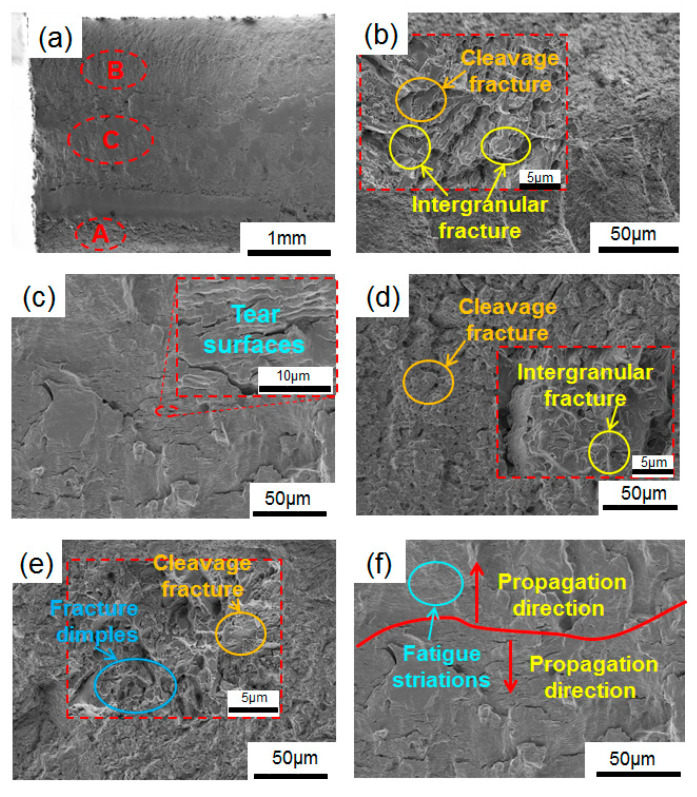
Fracture morphology of samples at the austenitization temperature of 1080 °C with 406 MPa (N_f_ = 1.97 × 10^4^): (**a**) macroscopic morphology; (**b**) the carburized layer; (**c**,**d**) the transition layer; (**e**,**f**) the core. A B and C indicate the SEM observation site of the carburized layer, the transition layer and core, respectively.

Figure 10, Figure 11 and Figure 12 present the fracture morphology of the specimens at different quenching temperatures at a stress level of 406 MPa. Overall, the macroscopic fracture processes of the three groups of specimens are similar and no obvious crack source areas can be observed in any of them, but the crack propagation area and the final fracture area can be seen. However, there are significant differences in the local fracture morphologies corresponding to different areas of the carburized layer, transition layer and core region of the specimen, reflecting that the gradient microstructure will affect crack propagation.

Figure 10a presents the fracture morphology of the specimens quenched at 1020 °C under a stress level of 406 MPa (N_f_ = 3.86 × 10^4^). From the macroscopic fracture morphology in Figure 10a, it can be seen that the overall fracture surface of the specimen is relatively flat. However, there are still obvious differences in the roughness and flatness of the fracture surfaces among the carburized layer, transition layer, and core. The carburized layer (Region A) and the transition layer (Region B) are relatively rough, whereas the core (Region C) is relatively flat. In addition, no obvious crack initiation zone is observed on the macroscopic fracture surface, but the crack propagation zone (Region C) and the final fracture zone (Regions A and B) can be identified. The final fracture zone of the specimen is located in the carburized layer and the transition layer, as shown in Figure 10b–d.

In the finally fractured carburized zone (Region A), a distinct “rock sugar-like” intergranular fracture feature can be observed, accompanied by local cleavage facets. The fracture mode of the specimen exhibits a mixed fracture form combining intergranular fracture and cleavage fracture. This is mainly attributed to the continuous distribution of carbides along the grain boundaries in the carburized layer. These carbides weaken the grain boundary bonding strength, making it easier for cracks to propagate along the grain boundaries, thus showing typical characteristics of intergranular brittle fracture, as shown in Figure 10b. In the finally fractured transition zone (Region B), as shown in Figure 10c, dimples and cleavage steps can be observed, which indicates a typical coexistence region of ductile microvoid aggregation and quasi-cleavage fracture. This indicates that after the crack propagates to this area, the material can, on the one hand, dissipate part of the energy through local plastic deformation, and on the other hand, undergo relatively rapid propagation along lath martensite or local interfaces. In addition, secondary cracks can be observed in this region, as shown in Figure 10d. Smooth tearing zones are visible around the cracks, while cleavage fracture traces are observed inside the cracks, indicating that this region is simultaneously affected by both local plastic tearing and brittle fracture mechanisms.

In the crack propagation zone of the core (Region C), micro-pores and dimples can be observed, indicating that a certain degree of plastic deformation occurs during the crack propagation process, as shown in Figure 10e. Additionally, some parallel and continuous fatigue striations can be observed in this region, as shown in Figure 10f, indicating that the crack did not form instantaneously but underwent a relatively stable initiation and propagation process under cyclic tensile–compressive stress loading.

In summary, the fracture morphology of the specimen austenitized at 1020 °C before quenching indicates that the core or near-core region of the specimens is an important area for the initiation and early stable propagation of fatigue cracks. The fatigue striations and local dimples observed in this region suggest a relatively stable propagation process under cyclic tension–compression loading. No unambiguous crack initiation site was directly observed. Cracks initiate at multiple sites in the core or near the core, gradually propagate toward the transition and carburized regions, and then exhibit intergranular brittle fracture accompanied by local quasi-cleavage fracture in the surface layer, finally leading to local instantaneous fracture.

Figure 11 shows the fracture morphology of the specimen quenched at 1050 °C under a stress level of 406 MPa. From the macroscopic fracture morphology in Figure 11a, it can be seen that the core (Region A) and the carburized layer (Region C) are relatively rough, while the transition layer (Region B) is relatively smooth. Moreover, a large area of tearing edges can be observed in the core. Similarly, no obvious crack initiation zone is observed on the macroscopic fracture surface.

The fracture morphology of the core area (Region A) is shown in Figure 11b. In the core area, alternating fracture traces and tearing marks accompanied by cleavage plane can be observed, which indicates that brittle fractures have occurred locally in this area under higher cyclic loads. These features indicate that fatigue crack propagation occurs in the core or near-core region, which implies that it might be the crack origin zone based on the apparent propagation direction, but the obvious crack initiation site cannot be directly observed from the current morphology of the fracture. Furthermore, it can be observed from Figure 11c that there is a large area of smooth tear surface in the core region, reflecting that the crack exhibited certain rapid propagation characteristics when extending through locally weak zones. This is attributed to the presence of two types of lath martensite with different sizes and spacings in the core of the specimen, quenched at 1050 °C, presenting an obvious ‘bimodal’ heterogeneous microstructure. Additionally, the hardness inhomogeneity in the core is relatively large. This local mismatch between microstructure and hardness reduces the compatibility of plastic deformation caused by cyclic loading and makes it easier to result in stress concentration in the core or near-core zone, thereby promoting the preferential initiation and propagation of microcracks along relatively weak areas.

Figure 11d shows the fracture morphology of the transition layer (Region B). Extensive tear surfaces can also be observed in this region. Higher magnification view reveals typical fatigue striation characteristics, indicating that the propagation stage of cracks extending outward from the core occurs in the region. It retains certain traces of fatigue propagation and also exhibits a tendency for local rapid crack propagation caused by the gradient change in the internal microstructure of the material. Figure 11e presents the fracture morphology of the carburized layer. A large area of tear planes is also visible in this region, displaying obvious roughening characteristics. The high-magnification morphology map shows intergranular fracture accompanied by dimple fracture (Figure 11f), indicating that rapid fracture occurs under cyclic loading when cracks initiate near the core region and gradually propagate through the transition layer to the carburized layer, ultimately forming a final fracture zone dominated by intergranular fracture with some ductile fracture features.

In summary, for the specimen austenitized at 1050 °C before quenching, the fracture characteristics reflect that the fracture mode of the aviation gear steel is a mixed fracture mode involving intergranular fracture, cleavage fracture and local ductile features. Although the apparent crack propagation direction suggests that the crack origin may be located in the core or near-core region, no clear crack initiation site was directly observed. The heterogeneous microstructure and uneven hardness distribution in the core maybe weaken the stability of crack propagation by promoting local deformation incompatibility and stress concentration in the core or near-core region. Therefore, under the same cyclic loading conditions, its overall fatigue performance is inferior to that of the specimen austenitized at 1020 °C.

Figure 12 illustrates the fracture morphology of the sample austenitized at 1080 °C under a stress level of 406 MPa. From the macroscopic fracture morphology in Figure 12a, it can be seen that the overall fracture surface of the specimen is relatively rough, and the roughness of the carburized layer (Area A) is relatively large, while the core (Area C) is relatively smooth. The final fracture is located in the carburized layer. Additionally, no obvious crack initiation zone is observed on the macroscopic fracture surface as well.

Figure 12b shows the morphology of the carburized layer after fracture. The fracture surface exhibits poor flatness. At higher magnification, an obvious intergranular fracture can be observed, accompanied by cleavage steps and blocky brittle spalling features. The fracture mode of the specimen is a mixed fracture pattern combining intergranular fracture and cleavage fracture. This is mainly attributed to the presence of numerous carbides distributed along the grain boundaries in the carburized layer, which weakens the grain boundary bonding strength and thus promotes crack propagation along the grain boundaries. During austenitizing at 1080 °C before quenching, although the number of carbides decreases, their size tends to coarser and their contours become irregular, making it easier to induce brittle delamination of the local material.

Figure 12c,d shows the morphology of the transition layer (Area B) after fracture. It can be observed that this region is dominated by cleavage fracture accompanied by intergranular fracture, indicating that under the influence of the gradient microstructure, this region exhibits a stronger tendency for rapid propagation of brittle fracture. In addition, fracture morphology features similar to those of the 1050 °C specimen can also be observed in the transition layer, namely the presence of large tear surfaces but without obvious fatigue striations, suggesting that the stable fatigue propagation characteristics in this region are weakened, and cracks propagate quickly along weak interfaces under the action of cyclic loading. The fracture morphology of the core is shown in Figure 12e,f. In this region, local micro-pores, dimples and tearing ridges can be observed, indicating that the outward crack propagation is accompanied by local plastic deformation and undergoes an initiation and propagation process under cyclic loading. Furthermore, fatigue striations and their propagation direction under cyclic tensile–compressive loading can be observed in Figure 12f.

Compared with the specimen austenitized at 1020 °C before quenching, the residual carbides in the core are further reduced after austenitization at 1080 °C before quenching, the lath martensite coarsens, and the overall hardness of the core decreases. Consequently, the load-bearing capacity of the specimen is weakened, making it easier for cracks to initiate at multiple sites in the core or near-core region and propagate outward more quickly. The smaller effective case depth, the lower hardness level of the core and the coarser microstructural features formed at the higher austenitization temperature may jointly reduce the inhibitory effect of the gradient microstructure on crack propagation, resulting in the lowest fatigue life and fitted fatigue strength for the specimen austenitized at 1080 °C before quenching.

## 4. Conclusions

In this work, the fatigue property of the aviation gear steel 15Cr14Co12Mo5Ni2 was investigated by means of the high-frequency fatigue test at high-temperature carburizing, and the microstructure and fatigue fracture mechanism of the specimens at three selected austenitization temperatures before quenching were analyzed in order to understand the influence of the austenitization temperature on its fatigue resistance. The main conclusions could be obtained as follows:

(1) After high-temperature carburizing and quenching, the aviation gear steel samples form a gradient microstructure from the carburized layer to the core. With the increase in the austenitization temperature, the carbide content in both the carburized layer and the core of the sample decreases, and the main carbide types are M_23_C_6_ and M_7_C_3_.
(2) The hardness of the aviation gear steel quenched at the three different temperatures all exhibits a gradient distribution characteristic, gradually decreasing from the surface to the core. The surface hardness of the samples quenched after austenitizing at 1020 °C and 1050 °C is higher than that of the sample quenched at 1080 °C, and the core hardness of the sample quenched at 1020 °C is the highest and the distribution is uniform. The effective carburized layer depth gradually decreases with the austenitization temperature before quenching increases, and their values are about 0.65 mm, 0.60 mm and 0.50 mm, respectively.
(3) Under the same austenitization temperature before quenching, the fatigue life of the aviation gear steel decreases as the stress level increases. The fatigue life of the aviation gear steel at the three austenitization temperatures is all higher than 10^6^ cycles at the low stress level of 203 MPa. Within the selected temperature range in this paper, the fatigue life decreases with increasing austenitization temperature at the stress level of 406 MPa, while at the higher stress levels of 609 MPa and 812 MPa, the fatigue life decreases firstly and then increases with the increasing austenitization temperature. The fitted fatigue strengths of 15Cr14Co12Mo5Ni2 gear steel at a given fatigue life of 10^6^ cycles under the austenitization temperatures of 1020 °C, 1050 °C and 1080 °C are 192 MPa, 183 MPa and 158 MPa, respectively.
(4) For the specimens at the three austenitization temperatures before quenching, no obvious crack source area could be directly observed from the fracture morphology of the specimens. The crack origin was inferred to be located in the core or near-core region according to the apparent crack opening and propagation features and the distribution of fatigue striations, and the cracks have a tendency to propagate outward from the core zone of the specimen, and the final fracture positions are all in the carburized layer of the specimen. As the austenitization temperature increases, the fracture morphology exhibited a stronger tendency toward brittle fracture features and the crack propagation rate from the core outward accelerates. The fracture mechanism of 15Cr14Co12Mo5Ni2 gear steel at the austenitization temperatures of 1020 °C was a mixed mode of intergranular and cleavage brittle fracture. At the austenitization temperature of 1050 °C, the fracture mode of the aviation gear steel was mainly intergranular fracture accompanied by ductile fracture. When the austenitization temperature is raised to 1080 °C, the fracture mode is predominantly cleavage fracture, along with intergranular fracture and local ductile fracture.

## Figures and Tables

**Table 1:** 15Cr14Co12Mo5Ni2W steel chemical composition (wt.%).

C	Cr	Co	Mo	Ni	W	V	Fe
0.13	13.89	12.49	4.61	1.99	0.62	0.61	Bal

**Table 2:** The content of each phase in different regions at different austenitization temperatures (%).

Phase	1020 °C			1050 °C			1080 °C		
	Carburized Layer	Transition Zone	Core Zone	Carburized Layer	Transition Zone	Core Zone	Carburized Layer	Transition Zone	Core Zone
Martensite	70.3	92.1	94.9	78.1	90.7	96.8	80.1	91.5	98.3
RA	0.2	0.4	0.1	0.1	0.4	0.1	0	0.4	0.5
Cementite	1.3	0	0.1	0.6	0.4	0	0.2	0.1	0
M_23_C_6_	5.4	6.6	4.5	3.7	5.9	2.3	16.5	5.8	0.9
M_7_C_3_	22.9	0.9	0.4	17.5	2.5	0.7	3.2	2.2	0.3

**Table 3:** The S–N fatigue test results for untreated initial specimens and those treated at different quenching temperatures.

	Stress (MPA)											
	203			406			609			812		
Initial specimens	35,000	27,000	36,000	14,600	16,000	15,000	1600	1400	1000	900	800	750
1020 °C	553,700	Run out	Run out	76,800	32,600	38,600	12,700	14,300	26,900	4700	5300	7800
1050 °C	Run out	Run out	Run out	62,700	9600	34,300	6200	4800	6100	1400	2400	2500
1080 °C	Run out	Run out	Run out	19,700	16,400	15,700	9200	8500	3800	3800	3100	3500

## Data Availability

The original contributions presented in this study are included in the article. Further inquiries can be directed to the corresponding authors.
